# Multiple cytokine analysis of aqueous humor in uveitis with or without secondary glaucoma

**DOI:** 10.1186/s12886-024-03691-4

**Published:** 2024-10-15

**Authors:** Junyan Xiao, Chan Zhao, Gangwei Cheng, Hang Song, Yang Zhang, Meifen Zhang

**Affiliations:** 1 Department of Ophthalmology, Peking Union Medical College Hospital, Chinese Academy of Medical Sciences and Peking Union Medical College, Beijing, China; 2 Department of Ophthalmology, Eye and ENT Hospital of Fudan University, Fudan University, Shanghai, China; 3 Shanghai Key Laboratory of Visual Impairment and Restoration, Eye and ENT Hospital of Fudan University, Fudan University, Shanghai, China; 4 National Health Commission (NHC) Key Laboratory of Myopia (Fudan University), Key Laboratory of Myopia, Chinese Academy of Medical Science, Shanghai, China

**Keywords:** Aqueous humor, Pro-inflammatory cytokines, TGF-β1, Uveitic glaucoma, Timing of surgery

## Abstract

To assess the level of both pro-inflammatory and anti-inflammatory cytokines in the aqueous humor (AH) of patients suffering from uveitis, with or without coexisting glaucoma, and compare them with patients diagnosed with primary open-angle glaucoma (POAG) and those with age-related cataract (ARC). By using Luminex xMAP® multiplex assays analyses, we assessed levels of 11 cytokines and chemokines, and compared them across groups, including uveitis-secondary glaucoma (USG) (*n* = 16), uveitis without glaucoma (UwoG), (*n* = 16), POAG (*n* = 16), and ARC (*n* = 16) to explore the correlation between these cytokines and the presence of uveitis, as well as intraocular pressure (IOP). Pro-inflammatory factors MCP-1, MIP-1β, IL-6, IL-8, and transforming growth factors TGF-β1 and TGF-β2 were significantly elevated in the AH of USG eyes. In the case of enhanced anti-inflammatory in the perioperative period, the pro-inflammatory factors remained notably elevated in the USG group compared to the UwoG group (*P* < 0.01). The levels of IL-6, IL-8, and MCP-1 in the AH of the USG group and POAG group had the same trend, which markedly surpassed those of the ARC group (*P* < 0.01). Significantly increased levels of MCP-1, MIP-1β, IL-6, IL-8, TGF-β1, and TGF-β2 were found in the AH of USG patients, implying a potential role for these mediators in the progression of glaucomatous manifestations within patients with uveitis. Besides the analysis revealed no discernible statistical disparity in cytokine concentrations within the AH of USG eyes whether the preoperative baseline IOP was greater than 30 mmHg or not, indicating that the safety of antiglaucoma surgery in USG patients even with baseline high IOP.

## Introduction

Disruption of the blood-retinal barrier can trigger retinal or uveal disease and lead to changes in the biochemical or protein composition of the aqueous humor (AH). Uveitis, recognized as an autoimmune disorder, the involvement of T lymphocytes, with a particular emphasis on Th cells and the cytokines they release, is crucial to understanding its underlying pathological mechanisms [1]. Uveitis-secondary glaucoma (USG), represents a frequent and serious complication of uveitis, potentially arising from various forms of ocular inflammation. Reports indicate that there is an increase in various biologically active molecules in the AH of uveitis patients, as detected by sophisticated bead immunoassays [2–8], such as monocyte interferon-γ (IFN-γ), chemoattractant protein-1 (MCP-1), interleukin-6 (IL-6) and interleukin-8 (IL-8) were increased in idiopathic uveitis AH; IFN-γ was detected elevating in anterior uveitis and panuveitis group; active intermediate uveitis is characterized by elevated levels of IL-6, IL-8, activator of normal T cell secretion (RANTES), and MCP-1. The sample sizes detected in these previous studies were small, and there have been no documented accounts examining various cytokines and their related background factors in the AH of USG patients.

While certain individuals afflicted with uveitis may experience angle-closure glaucoma, it is open-angle glaucoma that is predominantly common in USG. Approximately one in three adult sufferers of USG require surgical intervention [9]. A significant number of researchers hypothesize that secondary open-angle glaucoma is due to long-term alterations in the drainage route of the trabecular meshwork (TM). These alterations involve the buildup of extracellular matrix (ECM), variations in ECM protein composition, and a reduction in trabecular endothelial cells, which eventually result in diminished phagocytosis and modified protein degradation processes [10, 11]. An alternative explanation for elevated intraocular pressure (IOP) involves the physical blockage of the drainage channel due to accumulated debris and cells [10–12] In addition, studies have shown that excessively IOP elevated may also lead to damage to the blood-aqueous humor barrier, thereby promoting an influx of inflammatory cells and substances associated with inflammation into the AH. And the concentration of pro-inflammatory factors is positively correlated with IOP, suggesting that IOP can potentially stimulate cytokine production, while increased cytokine concentrations could conversely influence the AH dynamics and lead to increased IOP [13, 14]. Therefore, the localized immune reactions could vary between individuals in USG or uveitis without glaucoma (UwoG), thereby influencing the varying drainage efficiencies through the TM.

Whether the pathogenesis of POAG is immune-related has also been controversial. The modulation of these localized immunological reactions could be influenced by both pro-inflammatory and anti-inflammatory signaling molecules, which are secreted by various immune and non-immune cells, playing a pivotal role in managing the inflammatory process. Over the past few years, discoveries have revealed that immune factors are one of the non-stress-dependent risk factors for optic nerve damage, and cytokines can participate in IOP elevation and apoptosis through multiple pathways, accelerating the progression of glaucoma. Proinflammatory cytokines, such as tumor necrosis factor-alpha (TNF-α), are mainly generated by helper T cells and macrophages to enhance inflammatory processes [15]. Treatments for inflammatory conditions frequently utilize monoclonal antibodies that target pro-inflammatory cytokines or their respective receptors. Additionally, proinflammatory cytokines might contribute to the breakdown of ECM and the control of IOP [16]. Transforming growth factor beta (TGF-β) can have both pro- and anti-inflammatory properties. It has the potential to transform an area of active inflammation into one characterized by tissue repair [16]. Furthermore, TGF-β1 and TGF-β2 might stimulate the synthesis of ECM within the anterior chamber and simultaneously hinder the breakdown of pre-existing ECM [17, 18]. The effect of surgery on USG patients is unsatisfactory and controversial [19], they are relatively more resistant to filtering surgery modalities compared with POAG [20, 21]. However, there is currently no literature reporting the difference in inflammation between POAG and USG and it is not clear which cytokines can significantly affect the outcome of filtration surgery.

AH is easier to collect than vitreous and can better reflect the microenvironment in the eye than blood. We suppose that variations in outflow rates could mirror changes within the IL-8, MCP-1, and TGF-β signaling routes. Consequently, analyzing the levels of these cytokines in AH within the eye of individuals with uveitis, whether or not they also have glaucoma, could shed light on the underlying mechanisms affecting the drainage system. This analysis might offer insights that guide therapeutic strategies and predict outcomes related to glaucoma progression among those with uveitis. The Luminex xMAP® technology enables multiplex testing with exceptional specificity, permitting the concurrent detection of numerous monoclonal antibodies linked to magnetically tagged beads, each bearing a distinct fluorescent marker. These diverse bead varieties are blended and maintained in suspension within a standard 96-well microplate. The objective of this research was to identify and analyze the cytokine profiles in uveitis patients with or without glaucoma and compare them with POAG and ARC groups.

## Materials and methods

### Subjects

This research received authorization from the Institutional Review Board of the Peking Union Medical College Hospital in Beijing, China, which followed the principles outlined in the Declaration of Helsinki. All patients provided a documented agreement following an explanation of the study’s details.

In this research, a total of 64 eyes from 64 patients were collected, which included 16 USG eyes and 16 POAG eyes with anti-glaucoma surgeries, and 16 UowG eyes and 16 ARC eyes with cataract surgeries. The categorization of uveitis in this study adhered to the guidelines established by the International Uveitis Study Group (IUSG), taking into account the latest updates as referenced [22]. The cohort of patients was treated at the Peking Union Medical College Hospital during the period spanning from June 2021 to January 2022.

To enhance the precision of diagnosis and reduce the likelihood of biases by confounding factors, specific Inclusion and exclusion protocols were implemented. The inclusion criteria of USG and POAG patients were as follows: (1) conform to the diagnosis of USG or POAG; (2) patients over 18 years old underwent modified CLASS or dual-access surgery in our hospital; (3) no history of ocular trauma, and the history of intraocular surgery should be more than 1 year away from the current anti-glaucoma surgery; The exclusion criteria were detailed below: (1) suspicion of steroid-induced ocular hypertension; (2) shallow anterior chamber (the peripheral anterior chamber depth does not exceed 1/4 of the corneal thickness); (3) corneal opacity or opaque refractive media that could obstruct the assessment of the optic nerve; (4) patients with blindness in the other eye.

The inclusion criteria of UowG and ARC patients were as follows: (1) undergone cataract surgery at our hospital and have no active inflammation (anterior chamber cells ≥ 0) for three months prior to AH collection; (2) No alterations were observed in the optic nerve, and they had not been administered any form of IOP-lowering medication, whether topical or systemic, throughout their medical history; (3) IOP was consistently remained at or below 21 mmHg; (4) no history of other eye diseases, eye trauma, or eye surgery. The exclusion criteria were detailed below: patients with blindness in the other eye. In cases where patients received surgical treatment on both eyes, only the initial eye operated on was considered for inclusion. All patients were generally in good health, free from systemic illnesses including diabetes, infectious diseases, history of malignant tumors, serious mental and psychological diseases, etc., and the follow-up data were complete.

### Aqueous humor collection

For patients with USG or POAG, the collection of AH should be performed after anti-glaucoma surgery to make a scleral flap (before breaking the blood-aqueous humor barrier). In patients with UwoG or ARC, the collection of AH is performed after a clear corneal incision during cataract surgery. Each patient provided an AH sample ranging from 70 to 100 µL, collected via paracentesis into the anterior chamber using a 1 mL syringe and a 30-gauge needle. Post-collection, samples were promptly stored at -80 °C for subsequent analysis.

### Luminex xMAP® multiplex assays

The cytokines examined in this research were chosen for their implicated functions in ocular inflammation and the accessibility of detection kits. In this experiment, the Human XL Cytokine Premixed Kit (FSCSTM18-08, R&D Systems, USA) and TGF-β Premixed Kit (FSCSTM17-03, R&D Systems, USA) were utilized following the producer’s guidelines to identify cytokines in four groups of AH samples. Its detection concentration range is large, which can be accurately quantified to pg/ml, thus reducing the error [23]. Human XL Cytokine Premixed Kit contains IFN-γ, TNF-α, IL-6, IL-8, interleukin-10 (IL-10) and normal T cell secretion activator (RANTES), MCP-1, macrophage inflammatory protein-1β (MIP-1β) factors; TGF-β Premixed Kit contains different isoforms of TGF-β, namely TGF-β1, TGF-β2, and TGF-β3. In the TGF-β assay, samples were acidified and activated using the Sample Activation Kit1 activation kit from R&D systems.

Cytokine benchmarks were progressively diluted at a ratio of 1:3, while the AH samples were adjusted to a 1:2 ratio using the provided diluents. A total volume of 50 µL from the diluted samples was employed. The Luminex200 instrument, manufactured by Luminex Corp. in Austin, Texas, was used to measure the fluorescence intensity, which was then translated into cytokine concentrations as per the manufacturer’s directions, employing xPONENT3.1 software for detailed analysis. Due to the scarcity of the available AH, each sample was subjected to a single measurement.

### Statistical analysis

Statistical analyses were performed with SPSS software, version 23.0. Data about visual acuity, obtained from medical records, were transformed into logMAR values. Comparative analyses between the USG, POAG, UowG, and ARC groups were performed using ANOVA or the Mann–Whitney U test for continuous data, and the Chi-square test was applied to categorical data. The ANOVA test was selected for analyzing data that adhered to a normal distribution, whereas the Mann–Whitney test was applied for data that deviated from normality. The Spearman rank correlation coefficient assessed relationships among study variables. A *p*-value below 0.05 denoting statistical significance.

## Results

### Demographic data

Table 1 presents the demographic details of the patients. It indicates that the mean age for the POAG and ARC groups was notably older compared to the USG group (*P* = 0.039; *P* < 0.001), and the difference was related to the natural onset time of uveitis. The average preoperative baseline IOP for the USG group was markedly greater compared to the POAG group (*P* = 0.001). However, there was no observed statistical variation in the quantities of glaucoma medications used between the groups. Clinical features and baseline uveitis management for the patients are detailed in Table 2. Among USG patients, the etiologies of uveitis included four cases of Posner-Schlossman Syndrome, three of Behçet’s disease (BD), one case each of Vogt-Koyanagi-Harada (VKH) Syndrome and Fuchs Syndrome, and seven instances with undetermined causes. Nine of 16 UwoG subjects had received steroids systemically, while only 3 of 16 cases were in USG patients. All patients in the USG group were in a quiescent phase or only had mild inflammation (indicated by anterior chamber flare and inflammatory cells < 0.5 +) within two weeks before surgery, and the average duration of inflammation control was 3.94 ± 3.47 months.

**Table 1:** Baseline characteristics and ophthalmologic history of the patients

Characteristic	USG	POAG	UwoG	ARC
Patients	16	16	16	16
Male/Female	9/7	8/8	5/11	7/9
Age (years)				
Mean ± SD	43.0 ± 13.3^*^	54.3 ± 12.8	44.5 ± 11.7	68.3 ± 11.7^†^
Range	20–65	29–71	26–66	43–86
IOP (mmHg)				
Mean ± SD	34.4 ± 12.8^*^	22.6 ± 9.7	15.1 ± 3.1	15.1 ± 1.5
Range	20.6–59	14–49	10–21.0	12.7–17.5
Anti-glaucoma medications				
Numbers, Mean ± SD	3.2 ± 0.8	2.7 ± 0.8	0	0
Range	2.0–4.0	1.0–4.0	0	0
β-blockers	14 (87.5)	8 (50.0)	0	0
Prostaglandin analogs	7 (43.8)	14 (87.5)	0	0
Carbonic anhydrase inhibitor	15 (93.4)	13 (81.3)	0	0
α_2-_receptor agonists	13 (81.3)	8 (50.0)	0	0
Others	2 (12.5)	0	0	0
Glaucoma treatment time (months)				
Mean ± SD	37.2 ± 31.7	68.9 ± 50.7	0	0
Range	1–120	4–180	0	0
Previous phacoemulsification	6 (37.5)	3 (18.6)	0	0
Pupil seclusion with peripheral iridectomy	7 (43.8)	3 (18.6)	0	0

*UwoG* Uveitis without glaucoma

^*^P < 0.05, USG vs POAG

^†^*P* < 0.05, USG vs ARC

**Table 2:** Clinical characteristics of patients with uveitis and treatment of baseline uveitis

Characteristic	USG (*n* = 16)	UwoG (*n* = 16)
Diagnosis		
Idiopathic uveitis	7 (43.8)	7 (43.8)
Bechet’s disease	3 (18.8)	1 (6.3)
Vogt-Koyanagi-Harada syndrome	1 (6.3)	5 (31.3)
Posner-Schlossman syndrome	4 (25.0)	0
Fuchs’ heterochromic iridocyclitis	1 (6.3)	3 (18.8)
Anterior segment complications		
Peripheral anterior synechia (< 180 degree)	7 (43.8)	1 (6.3)
Extensive posterior synechia	1 (6.3)	4 (25.0)
Pupil seclusion with peripheral iridectomy	1 (6.3)	1 (6.3)
Duration of uveitis (years)	7.9 ± 5.2	6.2 ± 4.3
Inflammation control duration (months)	3.94 ± 3.47	4.56 ± 1.28
Medication		
Topical steroid eye drops, n (%)	13 (81.3)	9 (56.3)
Oral steroid, n (%)	3 (18.8)	9 (56.3)
Interferon, n (%)	2 (12.5)	0

*UwoG* Uveitis without glaucoma

### Analysis of aqueous cytokines

Table 3 presents the average values and their respective ranges for the cytokines identified within the AH across the four patient cohorts. Significant disparities were observed in the USG group compared to the control groups for cytokines IL-6, IL-8, MCP-1, MIP-1β, TNF-α, TGF-β1, and TGF-β2. Since RANTES levels were undetectable in all participants, it was excluded from further analysis.

**Table 3:** Aqueous cytokine levels across four patient groups (pg/ml)

Cytokines	USG		POAG		UwoG		ARC	
	Mean ± SD	Range	Mean ± SD	Range	Mean ± SD	Range	Mean ± SD	Range
MCP-1	2425.15 ± 2044.13	73.38–6466.54	1585.90 ± 1401.70	1.91–4608.98	1004.81 ± 782.51	404.79–3373.85	521.65 ± 118.17	347.81–741.36
MIP-1β	95.93 ± 15.72	50.71–120.72	98.26 ± 21.21	50.71–137.43	82.11 ± 17.51	50.71–120.72	81.98 ± 29.75	23.53–120.72
IL-6	1197.21 ± 1287.199	1.17–3532.78	1142.82 ± 2077.84	2.81–8070.88	47.81 ± 72.20	6.02–268.48	19.61 ± 44.97	1.74–161.7
IL-8	143.94 ± 103.58	3.37–394.51	161.60 ± 185.46	0.4–660.42	26.81 ± 31.05	4.73–119.81	4.68 ± 2.11	1.87–10.13
IL-10	27.64 ± 29.23	7.69–98.63	14.25 ± 8.49	2.59–27.99	34.20 ± 60.46	5.14–204.2	12.13 ± 7.86	5.14–22.92
IFN-γ	12.92 ± 7.02	2.73–23.39	12.48 ± 4.28	4.85–20.68	14.89 ± 10.10	5.56–43.5	10.71 ± 3.91	5.56–17.97
TNF-α	4.87 ± 1.41	2.6–7.68	3.83 ± 0.86	2.6–5.12	4.28 ± 2.17	2.29–10.94	3.31 ± 1.00	1.37–5.12
TGF-β1	523.69 ± 474.78	111.89–1951.95	148.66 ± 83.93	25.96–261.53	153.64 ± 168.37	12.98–626.84	46.71 ± 23.93	12.98–92.67
TGF-β2	1484.65 ± 827.57	387.33–3470.67	867.96 ± 642.77	299.61–2726.22	922.57 ± 415.91	363.63–1852.44	731.56 ± 223.13	377.43–1123.5
TGF-β3	38.31 ± 43.82	14.92–131.72	14.92 ± 0	14.92–14.92	20.24 ± 7.52	14.92–25.55	14.92 ± 0	14.92–14.92

*UwoG* Uveitis without glaucoma

**Fig. 1:**
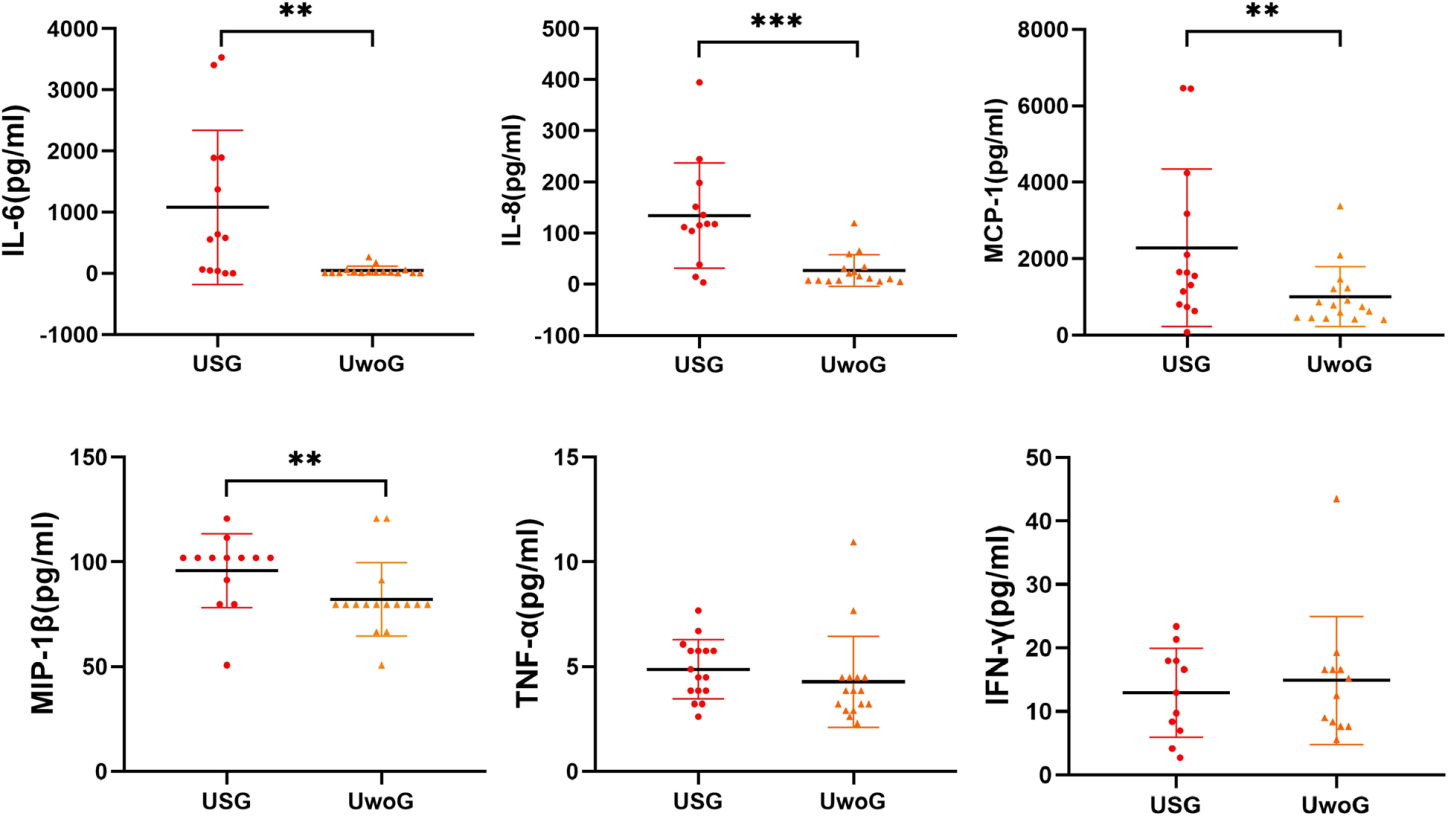
Comparison of six pro-inflammatory cytokines in the USG group and the UwoG group. UwoG: Uveitis without glaucoma; The ANOVA test was applied to TNF-α and IFN-γ due to their variance uniformity, whereas the Mann–Whitney U test was selected for the remaining cytokines to evaluate the concentration discrepancies between groups; A *p*-value of less than 0.05 was set as the threshold for statistical significance, with *P < 0.05, **P < 0.01, ***P < 0.001

Figure 1 illustrates the comparative analysis of six pro-inflammatory cytokines in the AH between patients with USG and UwoG. IL-6, IL-8, MCP-1, and MIP-1β levels in the USG group were markedly elevated with respective *p*-values of 0.001, less than 0.001, 0.008, and 0.007, surpassing those in the UwoG group. In contrast, no significant variation was noted in the levels of IFN-γ and TNF-α between the two groups.

**Fig. 2:**
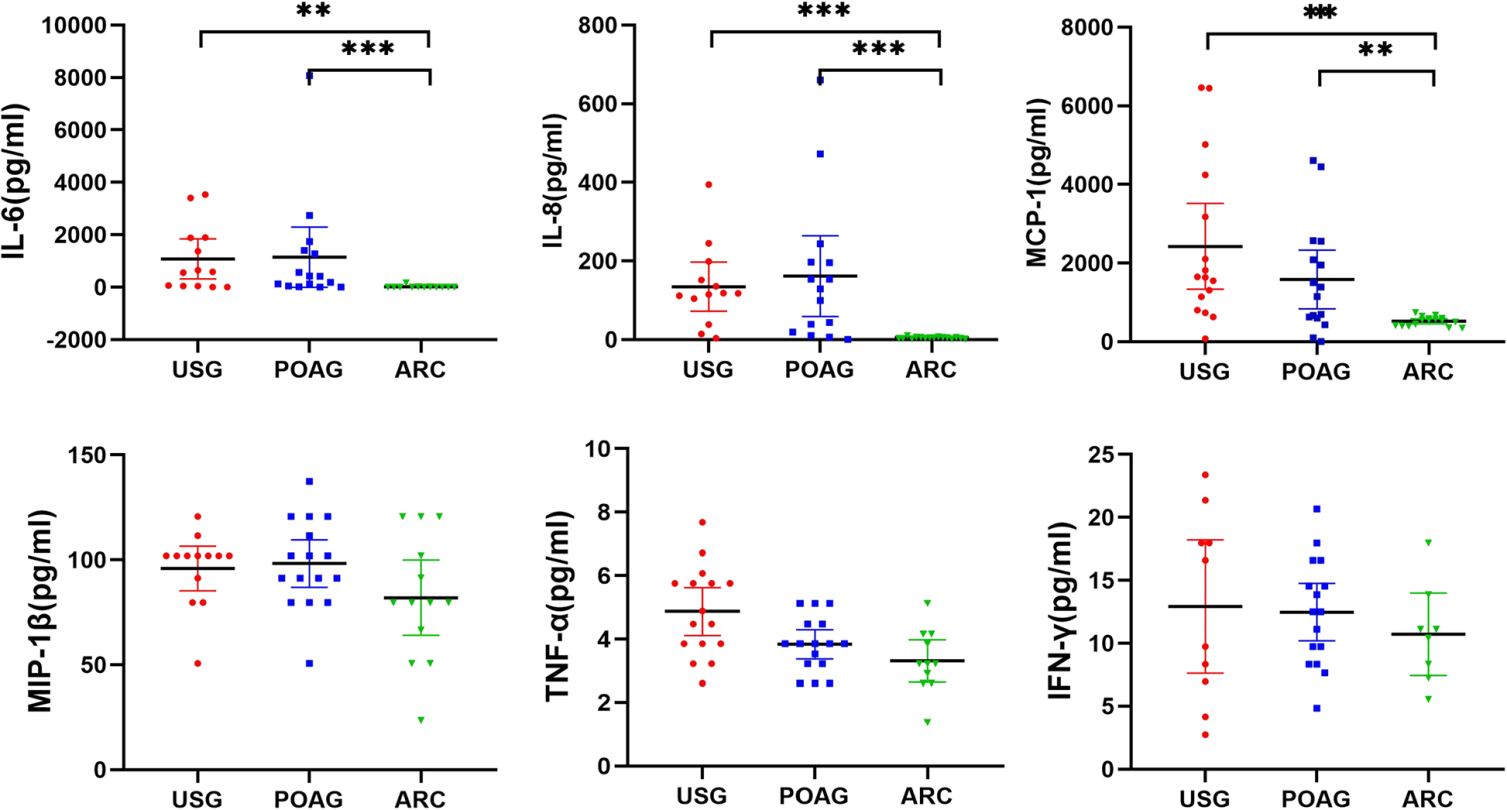
Comparison of six pro-inflammatory cytokines in the USG group, POAG group and ARC group. Except for TNF-α and IFN-γ, which conformed to the homogeneity of variance, the ANOVA test was used. The Mann–Whitney U test was employed to assess variations in cytokine concentrations between the two groups for all other factors. Statistical significance was determined at the *p* < 0.05 level, with *P < 0.05, **P < 0.01, ***P < 0.001

Figure 2 indicates no significant variance in the levels of six pro-inflammatory cytokines when comparing the USG and POAG groups. Nonetheless, as compared to the ARC group, the USG and POAG groups exhibited notably elevated IL-6 (*P* = 0.001; *P* < 0.001), IL-8 (*P* < 0.001; *P* < 0.001), and MCP-1 (*P* < 0.001; *P* = 0.003) levels respectively.

**Fig. 3:**
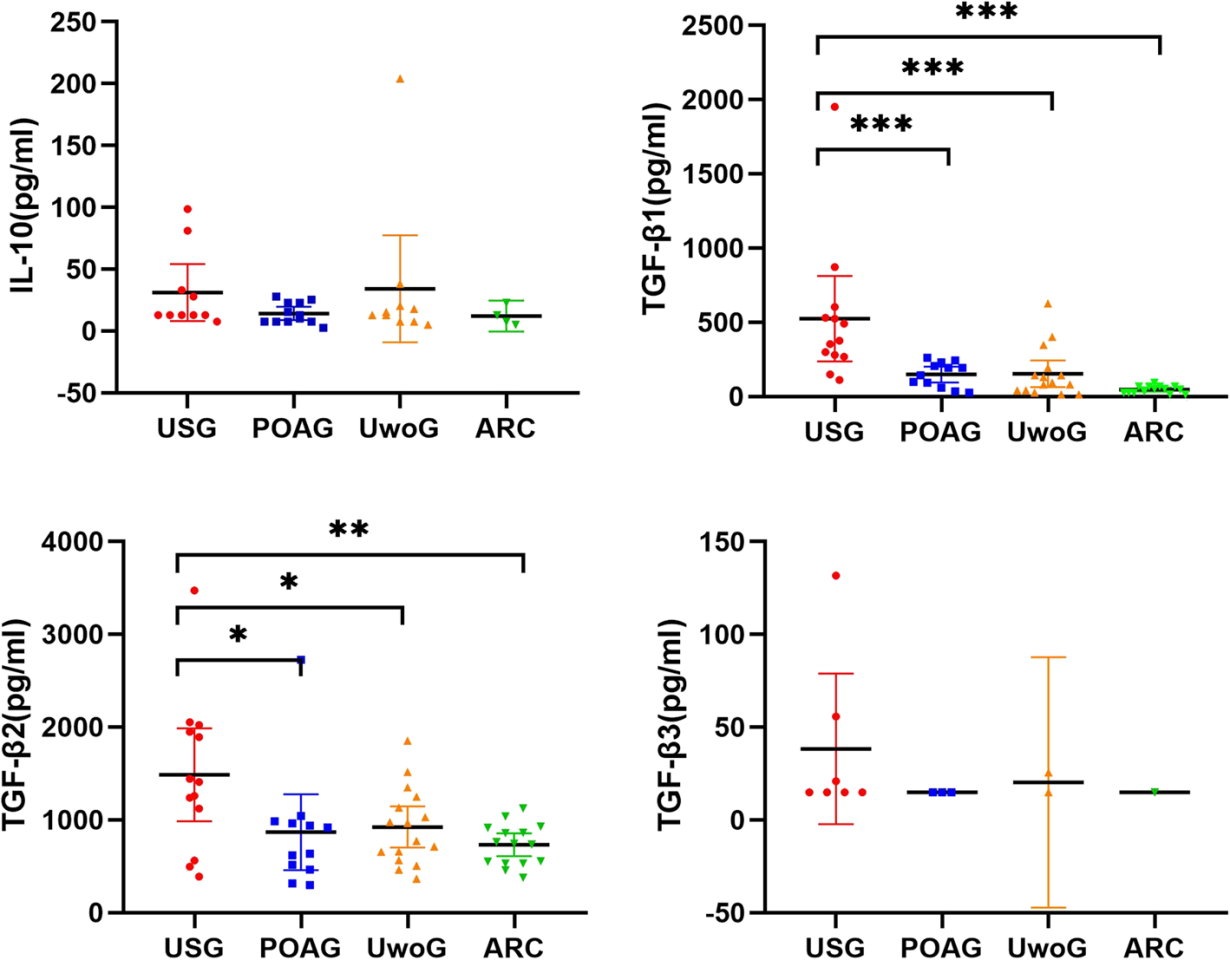
Comparison of IL-10 and TGF-β family cytokines among the four groups of patients. UwoG: Uveitis without glaucoma; The Mann–Whitney U test was applied to evaluate the disparity in cytokine levels between the two groups; *P* values ≤ 0.05 deemed to indicate statistical significance, with *P < 0.05, **P < 0.01, ***P < 0.001

In an assessment encompassing all four groups of patients. Figure 3 delineates that USG patients possessed considerably higher levels of TGF-β1 and TGF-β2 (TGF-β1: all *P* < 0.001; TGF-β2: *P* = 0.022; *P* = 0.045; *P* = 0.004), outstripping the concentrations found in the other three groups. TGF-β3 demonstrated an analogous trend but was not statistically different because most of the TGF-β3 were below the lowest detectable value (63.21 pg/mL). There was no statistically significant variation in IL-10, an anti-inflammatory cytokine, across the four groups.

### Correlation of cytokine levels with baseline IOP and age

**Fig. 4:**
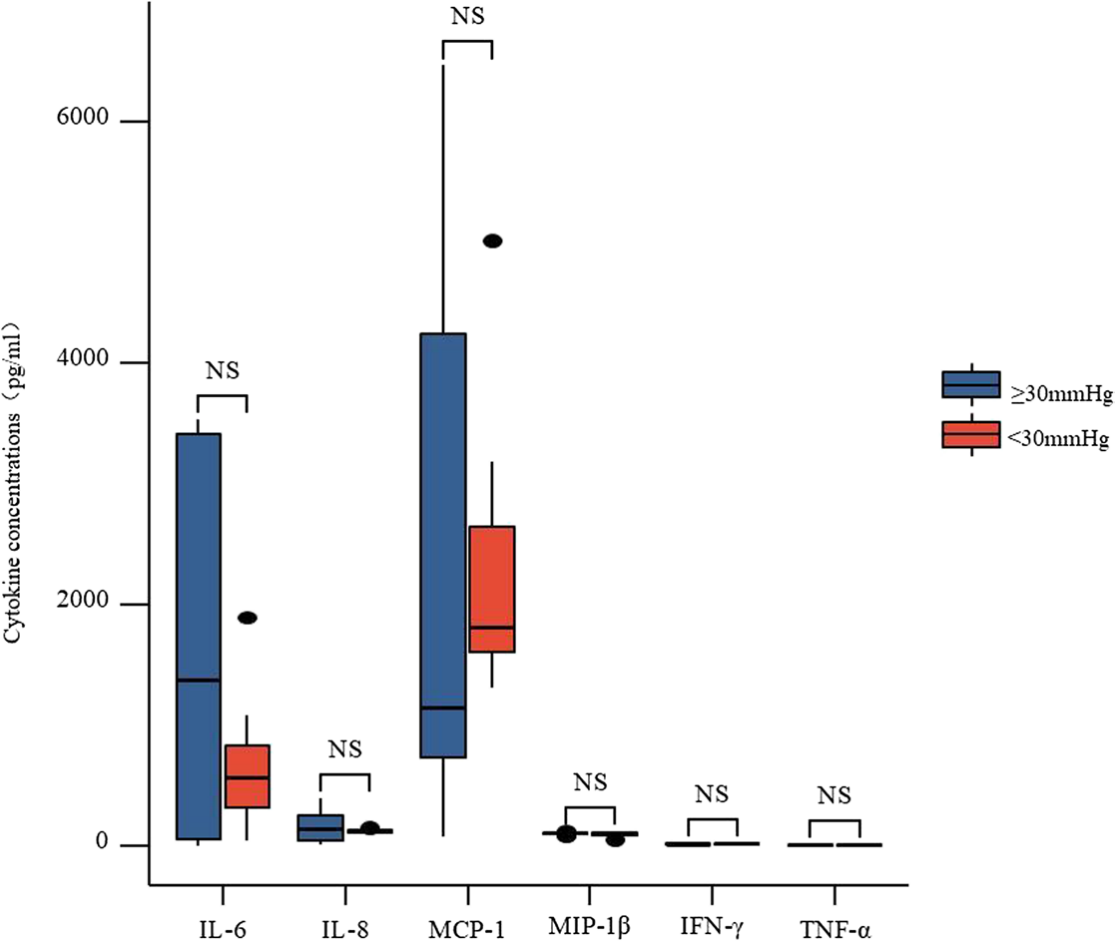
Aqueous cytokines with preoperative baseline IOP ≥ 30 mmHg and < 30 mmHg in USG eyes. NS: The difference is not statistically significant

Patients were categorized into two groups distinguished by their preoperative IOP: those with IOP ≥ 30 mmHg and those with IOP < 30 mmHg. Figure 4 illustrates that there was no significant variation in the levels of the six pro-inflammatory cytokines between the two groups of samples. Additionally, the cytokine correlation within the USG and UwoG groups did not exhibit substantial differences related to age.

### Correlation between cytokines

Table 4 displays the relationships between the levels of chemokines, growth factors, and cytokines across all participants. It reveals that TGF-β1 and MCP-1 had positive associations with TNF-α, IL-6 and IL-8. No negative correlations were identified among the different cytokines.

**Table 4:** Correlations between chemokines, growth factors, and pro-inflammatory factors

	MCP-1		IL-6		IL-8		IL-10		TNF-α		TGF-β1	
	r	*P*	r	*P*	r	*P*	r	*P*	r	*P*	r	*P*
MCP-1	——	——	0.729	< 0.001^*^	0.819	< 0.001^*^	0.443	0.005^*^	0.486	< 0.001^*^	0.662	< 0.001^*^
IL-6	0.729	< 0.001^*^	——	——	0.833	< 0.001^*^	0.281	0.083	0.220	0.097	0.642	< 0.001^*^
IL-8	0.819	< 0.001^*^	0.833	< 0.001^*^	——	——	0.199	0.225	0.316	0.016^*^	0.694	< 0.001^*^
IL-10	0.443	0.005^*^	0.281	0.083	0.199	0.225	——	——	0.369	0.021^*^	0.222	0.200
TNF-α	0.486	< 0.001^*^	0.220	0.097	0.316	0.016^*^	0.369	0.021^*^	——	——	0.429	0.002^*^
TGF-β1	0.659	< 0.001^*^	0.662	< 0.001^*^	0.694	< 0.001^*^	0.222	0.200	0.429	0.002^*^	——	——

^*^P < 0.05

## Discussion

The research uncovers notable changes in cytokine concentrations within the AH of USG patients when juxtaposed against three other groups, thereby substantially enhancing our comprehension of the AH’s role in USG patients. In the current study, even in the preoperative use of topical combined with systemic corticosteroids for anti-inflammatory, the proinflammatory factors in the AH of the USG group remained markedly elevated compared to the UwoG cohort. Secondly, the AH in both the USG and POAG groups exhibited similarly increased concentrations of pro-inflammatory factors like IL-6, IL-8, and MCP-1, which were considerably higher compared to the ARC group. These cytokines may contribute to the development of increased ocular pressure. Additionally, there is currently no guideline on the timing of anti-glaucoma surgery in eyes with USG. Our research stands as an initial investigation indicating no variance in cytokine expression levels in the AH of USG patients, regardless of whether they presented with high baseline IOP prior to surgery.

Many investigations have demonstrated that the occurrence and progression of uveitis are contributed from inflammatory mediators and chemokines. Studies have indicated that during the clinical quiescent stage of uveitis, the AH has higher levels of chemokines, with notably increased levels of MCP-1, MIP-1β, and IL-8, where MCP-1 is linked to post-cataract surgery inflammation relapse [24]. Ohira et al. [25] observed that an array of cytokines, comprising MCP-1, TNF-α, IL-6 and IL-8 were notably higher in the AH among patients afflicted with neovascular glaucoma. Nevertheless, due to the typically limited sample volume of AH, ranging from 50 to 150 μl, that can be obtained from USG patients, previous research on USG was scarce. This study, however, identified several cytokines that are intimately associated with USG, including IL-6, IL-8, MCP-1, MIP-1β, TNF-α, TGF-β1, and TGF-β2.

MCP-1 represents a typical pro-inflammatory molecule within the C–C chemokine family and also has fibrogenic effects, and is a potent chemotactic activator of human monocytes and macrophages. Elevated MCP-1 levels facilitate the mobilization and stimulation of mononuclear cells in uveitis eyes. Numerous investigations have confirmed the critical involvement of macrophage infiltration in the tissue damage associated with uveitis. The increase of MCP-1 in the AH can recruit macrophages to accumulate in the anterior chamber and TM, and macrophages are more likely to phagocytose inflammatory factors. The TM is blocked, causing diminished AH filtration and a rise in IOP. The more severe the local inflammatory response, the higher MCP-1 expressed, and the more obvious cell migration and inflammatory infiltration, which is consistent with the preoperative IOP and MCP-1 levels in the USG group were significantly elevated compared to the other three groups in this study.

MIP-1β, as the main factor produced by macrophages after stimulation, has the function of regulating other peripheral immune cells [26]. IL-8 is capable of being generated by macrophages as well as a variety of cells resident in the eye, and is an inflammatory factor with chemotaxis. The attraction of neutrophils by IL-8 has been noted in samples from the vitreous body affected by uveitis and other vitreoretinal diseases [27]. In addition, the inner retina and TM tissue produce endogenous IL-8 [28, 29]. Research indicates that IL-8 may suppress the function of RGC-5 cells through the induction of apoptosis [30]. In addition, ischemia hypoxia and elevated IOP can both induce the production of IL-8 [31], and the levels of IL-8 demonstrated a positive association with IOP, suggesting that elevated IL-8 can positively enhance the outflow resistance of AH [32]. MIP-1β and IL-8 are pro-inflammatory chemokines that recruit immune cells. Interestingly, in this study, both chemokines were highly expressed and at similar levels in the USG and POAG groups, significantly elevated than the ARC group, suggesting that the immune system has a significant effect on retinal nerve cells [33, 34]. Additionally, TGF-β and IL-8 showed a positive correlation, indicating that elevated levels of these cytokines may reflect a significant reduction in the AH outflow via the TM, and both inflammatory reactions are implicated in the development of both types of glaucoma.

Compared with primary angle-closure glaucoma, POAG has lower IOP, less intraocular ischemia and hypoxia, and less tissue damage. Thus, fluctuations in cytokine levels within the AH are likely a consequence of damage to the TM and optic nerve. Currently, the origin of IL-6 in the AH remains uncertain. However, evidence from clinical and animal studies indicates that IL-6 plays a substantial role in the development of numerous autoimmune conditions, such as rheumatoid arthritis [35]. IL-6 has been associated with a spectrum of central nervous system disorders, including nerve damage, neurodegenerative diseases, etc. [36]. The relationship between IL-6 and nerve cells is still debated, some scholars believe that IL-6 accelerates nerve cell death and causes neuroinflammation [37]; and apoptosis of nerve cells [38]. In addition, various research has documented variations in IL-6 levels within the AH of individuals suffering from POAG. For example, Borkenstein et al. [39] and Takai et al. [40] have demonstrated that IL-6 concentrations in the AH of patients with POAG are significantly lower than those found in a control group of cataract patients; Kuchtey et al. [28] reported no substantial variation in IL-6 concentrations in the AH when comparing POAG patients to the cataract control group. The above differences could potentially be attributed to the inclusion criteria and the size of the sample. This study found that the IL-6 levels in both the USG and POAG groups were notably elevated compared to the ARC group, and these levels demonstrated a significant positive correlation with IOP. Additionally, other studies have indicated that IL-6 possesses the ability to trigger the STAT3 signaling pathway, mediate the production of extracellular matrix [41], indicating that IL-6 is likely to mediate the increase in IOP by a certain mechanism. We, therefore, recommend perioperative hormonal therapy for patients planning antiglaucoma surgery to achieve both anti-inflammatory and optic nerve protection goals [42, 43].

T cells are considered to have a principal role in the pathogenesis of uveitis [44, 45]. RANTES functions as a chemokine that attracts T cells and CD4 + monocytes to antigenically challenging sites [46, 47]. In the present study, RANTES was below the lowest detectable value in all subjects, and the intraocular chemotactic activity of CD4 + T cells was not elevated, suggesting that patients in either the USG group or the UwoG group were in a period of relative quiescent stage in the context of strong perioperative anti-inflammatory efforts.

TGF-β is a cytokine with an immunomodulatory activity that can be secreted and expressed in many tissues, but its functions within the pathophysiology of USG remains not completely comprehended. Previous literature reported that TGF-β1 played a profibrotic role in fibroblasts, and TGF-β2 could promote the proliferation of extracellular matrix and fibroblasts [48]. Because TGF-β1 and TGF-β2 present in the AH are known to stimulate the contraction of TM cells during the initial phase and to induce TM fibrosis in the later stages, which leads to the increase of IOP [49], which can reflect the damage of the TM. In our study, the positive correlation between TGF-β1 and TGF-β2 and the baseline preoperative IOP also confirmed this. Therefore, we speculate that the increase in aqueous outflow resistance and IOP in USG patients is related to the concentration of TGF-β [49–51]. It’s intriguing to note that TGF-β can suppress T cell proliferation that is reliant on IL-2 by impeding the activation of IL-2 and transferrin receptors [52]. Beyond neuropeptides like α-stimulating melanocyte hormone and vasoactive intestinal peptide, TGF-β is also considered to play a role in sustaining the intraocular immunosuppressive environment [53], thus TGF-β is a multifunctional factor. It has been observed that inflammation in patients suffering from idiopathic uveitis is reduced with increasing TGF-β2 concentrations [2]; According to Min et al. [54] AH from patients with POAG showed an increase level in TGF-β2, whereas in USG (open-angle type), the TGF-β2 levels were found to be within normal limits. In the present research, it was observed that the USG group had markedly higher levels of both TGF-β1 and TGF-β2 in the AH compared to the other three groups. Secondly, TGF-β1 showed a positive correlation with TNF-α and MCP-1. The up-regulation of MCP-1 expression induced by the acyl-inositol 3-kinase signaling pathway was consistent. There are also animal experiments that found that TGF-β will increase responsively and antagonize the early production of IL-6 during the development of inflammation, thereby inhibiting the development of inflammation [55]. In light of these results, our belief is that the marked increase of TGF-β in USG eyes is related to its responsive antagonism of other significantly increased pro-inflammatory factors, and whether TGF-β occupies a central role in USG and its exact mechanism remains to be further elucidated.

Surgical intervention for glaucoma serves as a potent strategy to preserve vision in patients with USG, and there are no guidelines regarding the timing of surgery. As a preliminary study, we found that no variation was observed in cytokine levels within the USG group according to whether the preoperative baseline IOP was greater than 30 mmHg, suggesting that there is no substantial link between the initial IOP and the increased expression of pro-inflammatory factors in patient eyes. A high IOP in the affected eye does not necessarily imply surgery failure. Further future studies are essential to determine the specific timing and corresponding requirements for anti-glaucoma surgery in patients with uveitis.

This research has a few constraints. First, the sample size of our study was constrained, and each sample was assessed a single time due to the scarcity of AH volume. However, the comparative outcomes among the four groups were statistically robust and compelling; second, due to the limited sensitivity of the corresponding factor of the kit, some cytokine levels could not be measured, but it cannot be ruled out that they are altered in the disease. Third, because the surgical patients selected in this study are in the quiescent stage of inflammation or only have mild inflammation for treatment, further experimental exploration is needed for the changes of cytokines in the active stage of inflammation and the effects on cytokine expression before and after drug treatment.

In conclusion, this research has furnished an in-depth analysis of the diverse cytokine profiles in the AH of USG patients, while revealing their differences from the intraocular microenvironment of POAG. Our research marks the initial report indicating notably increased concentrations of IL-6, IL-8, MCP-1, MIP-1β, TGF-β1, and TGF-β2 in USG patients, suggesting that USG-affected eyes have elevated pro-inflammatory cytokines, damage to the TM and the disturbance of the extracellular matrix implicates a potential role for these cytokines in the progression of USG.

## Data Availability

Some or all data, models, or codes generated or used during the study can be obtained from the corresponding author upon request.
